# The Influence of Environmental Hypoxia on Hemostasis—A Systematic Review

**DOI:** 10.3389/fcvm.2022.813550

**Published:** 2022-02-18

**Authors:** Benedikt Treml, Bernd Wallner, Cornelia Blank, Dietmar Fries, Wolfgang Schobersberger

**Affiliations:** ^1^General and Surgical Intensive Care Medicine, Department of Anesthesiology and Critical Care Medicine, Medical University Innsbruck, Innsbruck, Austria; ^2^Department of Anesthesiology and Critical Care Medicine, Medical University Innsbruck, Innsbruck, Austria; ^3^Institute for Sports Medicine, Alpine Medicine and Health Tourism, Private University for Health Sciences, Medical Informatics and Technology UMIT, Hall i.T. and Tirol Kliniken GmbH, University Hospital Innsbruck, Innsbruck, Austria

**Keywords:** hypoxia, altitude, hemostasis, coagulation, fibrinolysis, thrombin generation

## Abstract

Humans have been ascending to high altitudes for centuries, with a growing number of professional- and leisure-related sojourns occurring in this millennium. A multitude of scientific reports on hemostatic disorders at high altitude suggest that hypoxia is an independent risk factor. However, no systematic analysis of the influence of environmental hypoxia on coagulation, fibrinolysis and platelet function has been performed. To fill this gap, we performed a systematic literature review, including only the data of healthy persons obtained during altitude exposure (<60 days). The results were stratified by the degree of hypoxia and sub-categorized into active and passive ascents and sojourns. Twenty-one studies including 501 participants were included in the final analysis. Since only one study provided relevant data, no conclusions regarding moderate altitudes (1,500–2,500 m) could be drawn. At high altitude (2,500–5,400 m), only small pathophysiological changes were seen, with a possible impact of increasing exercise loads. Elevated thrombin generation seems to be balanced by decreased platelet activation. Viscoelastic methods do not support increased thrombogenicity, with fibrinolysis being unaffected by high altitude. At extreme altitude (5,400–8,850 m), the limited data showed activation of coagulation in parallel with stimulation of fibrinolysis. Furthermore, multiple confounding variables at altitude, like training status, exercise load, fluid status and mental stress, prevent definitive conclusions being drawn on the impact of hypoxia on hemostasis. Thus, we cannot support the hypothesis that hypoxia triggers hypercoagulability and increases the risk of thromboembolic disorders, at least in healthy sojourners.

**Graphical Abstract:**
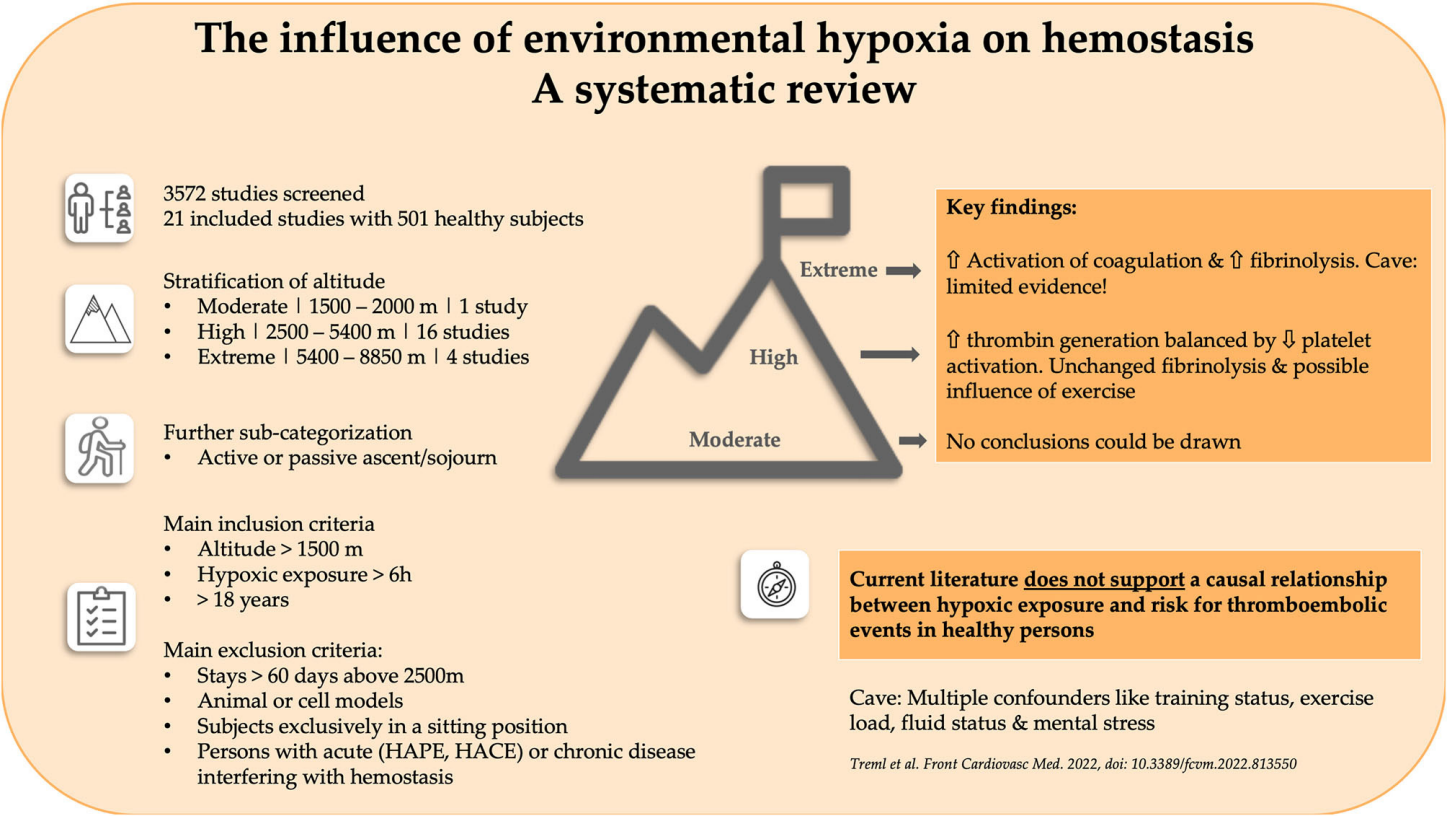


## Introduction

In the past few decades, a growing number of lowlanders have been sojourning at high altitudes for professional and leisure activities. The popularity of guided trekking tours at remote high-altitude regions remains high. However, some trekking tourists may suffer from lifestyle-related diseases, such as hypertension or obesity. Exposure to high altitudes, and the associated hypobaric hypoxia, induce a variety of physiological adaptations, a pivotal being the upregulation of hypoxia inducible transcription factors (HIFs) (1); maladaptation can also occur (e.g., acute mountain sickness), manifested as clinically relevant pathophysiological changes of the hemostatic system (2). These “coagulopathies” at high altitude mostly present as venous thrombosis or/and venous thromboembolism (VTE; deep venous thrombosis, pulmonary thromboembolism, mesenteric vein thrombosis, and cerebral venous sinus thrombosis [CVST]) (3–5). Cases of coronary stent thrombosis have even been reported, and cerebrovascular stroke occurring at high altitude was discussed in association with increased thrombogenicity (6). In the literature, a multitude of articles point to high altitude itself as an independent factor for adverse changes in the hemostatic system (7). Despite well-defined risk factors for VTE, little is known about the effects of high altitude on VTE risk in the general population; at present limited evidence exists to suggest a causal relationship. Various case reports have described patients who suffered from detrimental coagulation disorders at high altitude (8). A relationship between high altitude and CVST has long been suspected, based on anecdotal evidence from past case reports (9, 10). One study highlighted potential reversible risk factors for CVST occurring at high altitude, including smoking and dehydration. The increasing incidence of CVST has been associated with hypobaric hypoxia and strenuous exercise undertaken at peak summits. Pulmonary arterial embolism (PAE) is reported to be associated with high-altitude pulmonary edema (HAPE); however, the evidence indicates that PAE is not the cause, but rather the consequence, of HAPE. For all of these clinical findings, additional factors (e.g., extreme cold, exhaustion, a prolonged period of immobility in a tent, polycythemia and dehydration) were suggested to be contributory. A further confounding factor might be long-distance travel via aircraft, train, bus or car to remote mountain regions; such travel has been associated with the development of VTE (“travel thrombosis”) due to various factors (e.g., sitting in a cramped space during travel). Analysis of all existing reports on thromboembolism occurring at high altitude indicates that the majority of the patients suffered from preexisting medical conditions, i.e., inherent coagulation defects such as protein C (PC) and protein S (PS) deficiency, antiphospholipid syndromes and factor V Leiden mutation (5, 11, 12). Moreover, in patients with polycythemia vera residing at high altitude an association between altitude and an increased risk of thrombotic complications, most likely due to chronic increased HIF activation has been reported (13).

Despite this multitude of case reports and publications (albeit including small numbers of patients), there is no clear consensus regarding the risk of acquired hemostatic disorders at high altitude. This knowledge is crucial, because a growing number of people with and without preexisting diseases are traveling to high-altitude areas. At present, thorough scientific analysis of the influence of environmental hypoxia on coagulation, fibrinolysis and platelet function is lacking. In order to fill this gap, this systematic review aims to determine the influence of high-altitude hypoxia, typically encountered during trekking or mountaineering, on the hemostatic system. Chronic hypoxic exposure like in high-altitude dwellers or patients with congenital augmentation of hypoxia sensing are outside of the focus of this review.

## Methodology

### Protocol

To address the research question, a systematic literature review was performed. The method for identifying and including studies was consistent with the recommendations of the Cochrane Collaboration, as well as the guidelines of the Preferred Reporting Items for Systematic Reviews and Meta-Analyses Statement for Reporting Systematic Reviews (PRISMA).

### Study Selection and Eligibility Criteria

This systematic search included literature from PubMed and the Cochrane Library (central register of controlled trials), and considered studies published from 1950 up until the 1^st^ of July, 2021. We performed a broad and comprehensive search for articles and selected abstracts, titles and full texts using the following strategy: MeSh terms, i.e., “hypoxia,” “hypoxemia,” “altitude,” and “high altitude” were combined with OR and additionally combined with AND with the search block of “coagulation” OR “hemostasis” OR “fibrinolysis” (again, including text words and respective MeSh terms). Additionally, articles identified via manual search of the reference lists of included articles, as well as articles from grey literature (personal contacts of the authors), relevant to the topic were included.

### Inclusion and Exclusion Criteria

Studies were required to meet the following inclusion criteria: (1) minimum altitude of 1,500 m above sea level, where altitude was classified as moderate (1,500–2,000 m); high (2,500–5,400 m) or extreme (5400–8850 m); (2) active (exercise during ascent and/or a sojourn) and passive hypoxic exposure (hypoxic chamber, passive ascent); (3) study participants were males or females aged over 18 years; and (4) original full-text articles written in English.

The following exclusion criteria were applied: (1) studies using animals or cell models; (2) short-term hypoxic exposure <6 h; (3) long-term stays (>60 days) above 2,500 m, to exclude high-altitude natives/residents and prolonged job-related sojourns (e.g., working in a hut or for the military); (4) intermittent bouts of hypoxic exposure; (5) studies including subjects exclusively in a sitting position during hypoxic exposure, to exclude venous stasis (“travel thrombosis”) as a potential confounder; (6) patients with preexisting diseases possibly interfering with hemostasis; (7) studies on HAPE and high-altitude cerebral edema (HACE) patients [although studies with a clearly defined asymptomatic population (control group) were included for further analysis]; and 8) any form of medical treatment interfering with hemostasis.

### Data Extraction

The online tool Covidence (Covidence systematic review software, Veritas Health Innovation, Melbourne, Australia. www.covidence.org) was used for the screening, selection and classification of all articles. A total of 3,572 studies were identified in the initial search. In total, 58 duplicates were removed. The titles of the remaining articles were screened independently for suitability by two researchers (BT and BW). Any disagreements were resolved by discussion with a third author (WS). After screening 3,514 articles by title, 3,446 studies were excluded; the abstracts of the remaining 68 studies were screened for suitability. Of those, 48 were of further interest and their full texts were thus retrieved. One additional study was handselected. Ultimately, 21 studies (501 participants) were included in the final analysis (Table 1). A flow chart of the search process is illustrated in Figure 1. All selected studies were included in the qualitative analysis outlined in the next section.

**Table 1:** Overview of the 21 included studies (*N* = 501).

			**Maximum altitude**			
**Authors**	**Participants^**#**^**	**Study design**	**Moderate**	**High**	**Extreme**	**Outcome** **measurements**
	**(n)**		**1500–2500 m**	**2500–5400 m**	**5400–8850 m**	
Albrecht and Albrecht (14)	11^#^	Prospective cohort			6200	Standard coagulation parameters, blood count
Andrew et al. (15)	8	Prospective cohort			7625	Coagulation factors
Bartsch et al. (16)	11^#^	Prospective cohort		4559		Standard coagulation parameters, fibrinolysis
Bendz et al. (17)	20	Prospective cohort	2400			Coagulation factors, fibrinolysis
Coppel et al. (18)	28	Prospective cohort		4500		Thromboelastometry
Doughty and Beardmore (19)	6	Prospective cohort			5700	Standard coagulation parameters
Kicken et al. (20)	18	Prospective cohort		3883		Standard coagulation parameters, thrombin generation, platelet activation
Kicken et al. (21)	6	Prospective cohort		3775		Standard coagulation parameters, thrombin generation
Lehmann et al. (22)	10	Prospective cohort		4559		Blood count, platelet function
Maher et al. (23)	8	Prospective cohort		4400		Standard coagulation parameters
Martin et al. (24)	17	Prospective cohort		5300		Standard coagulation parameters, thromboelastometry
Modesti et al. (25)	31	Prospective cohort		5400		Blood count, thromboelastometry
Ninivaggi et al. (26)	30	RCT, 2 arms		3900		Standard coagulation parameters, fibrinolysis, thrombin generation
Pichler Hefti et al. (27)	34	RCT, 2 arms			6865	Standard coagulation parameters
Rocke et al. (28)	63	Prospective cohort		5200		Standard coagulation parameters, thromboelastometry, platelet function
Schaber et al. (29)	14^#^	Prospective cohort		4500		Standard coagulation parameters, thromboelastometry, thrombin generation
Sharma (30)	15	Prospective cohort		3000		Platelet function
Sharma (31)	61^#^	Prospective cohort		3660		Platelet function
Sharma and Hoon (32)	18^#^	Prospective cohort		3658		Platelet function
Singh et al. (33)	16^#^	Prospective cohort		3505		Standard coagulation parameters, fibrinolysis
Zafren et al. (34)	76	Prospective cohort		5340		Fibrinolysis

*N, number of participants; m, meters; RCT, randomized controlled trial*.

# *Only healthy study participants were included if data available for this group*.

**Figure 1:**
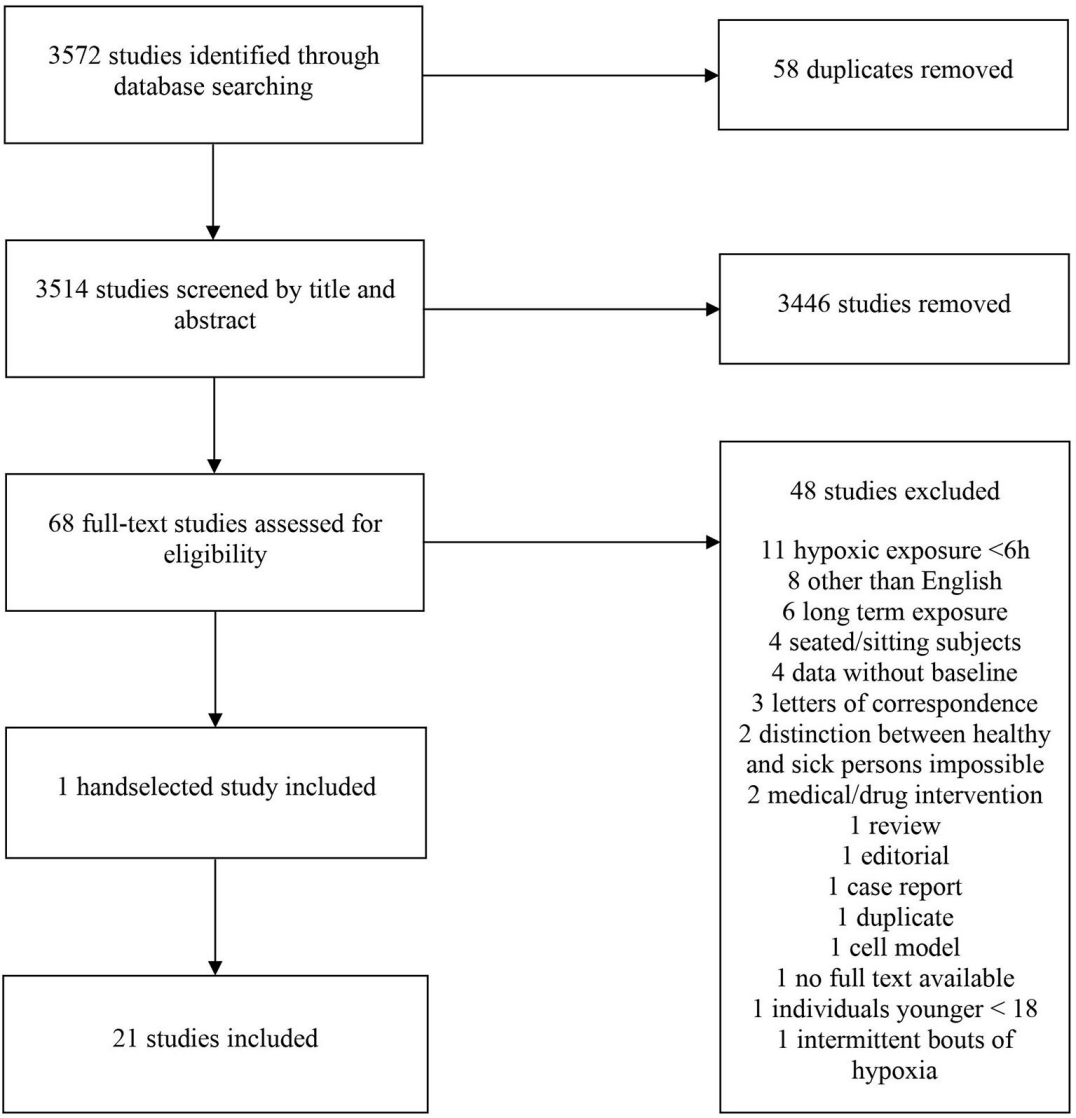
PRISMA flow chart of the search process.

### Quality Assessment and Risk of Bias

A modified Downs and Black tool was used to determine the quality of the articles according to a 13-point scale (35). Up to 7 points could be awarded depending on how the study reported its findings, as well as up to 2 points for external validity and up to 4 for internal validity (bias and confounding). The quality assessment score of each article was classified as follows: 11–13: Excellent; 9–10: Good; 7–8: Fair; and ≤ 6: Poor. The level of evidence was assessed using the Oxford Centre for Evidence Based Medicine (OCEBM) system (36). The OCEBM system is hierarchical, grading studies on a scale ranging from 1 (highest level of evidence) to 5 (lowest level of evidence), and including subsections for levels 1–3. Both reviewers (BT and BW) independently scored the articles and agreed on the final score for each following a discussion.

## Results

### Methodological and Reporting Quality

Two randomized controlled trials (26, 27) and nineteen prospective cohort studies were included in our review (14–25, 28–34). The Oxford Level of Evidence ranged from 2b to 4b and the Downs and Black Quality Assessment revealed that there were 3 studies of good quality (26, 27, 29), 8 of fair quality (14, 16, 20, 21, 24, 25, 28, 34) and 10 of poor quality (15, 17–19, 22, 23, 30–33). Details are provided in Supplemental Table S1.

### Characteristics of the Included Studies

The main findings of the included studies are summarized in Tables 2–4, stratified by the parameters measured and altitude categories. We included 1 study (17) (*n* = 20) conducted at moderate altitude (1,500–2,500 m), 16 (16, 18, 20–26, 28–34) (*n* = 422) conducted at high altitude (2,500–5,400 m) and four (14, 15, 19, 27) (*n* = 59) conducted at extreme altitude (>5,400 m).

**Table 2:** Changes of standard coagulation parameters, coagulation factors, red blood cells and platelet count during exposure to moderate, high and extreme altitudes (16 studies, *n* = 388).

**Authors**	**Maximum altitude [m]**	**Participants** **M/F, age [years]**	**Type of** **physical activity**		**Maximum duration**	**Parameter**	**Maximum change**	**Level of** **significance^†^**
			**Active**	**Passive**				
**Moderate altitude**								
Bendz et al. (17)	2400 (simulated)	20 M 20–40		Activities of daily living	8 h	F.VII (U/L)	+17%	*p* = 0.008
						F.VII AG (%)	−5%	*p* = 0.003
						PTF 1+2 (nM/L)	+250%	*p* = 0.008
						TAT complex (μg/L)	+820%	*p* = 0.04
**High altitude**								
Bartsch et al. (16)	4559 (terrestrial)	11 M 22–55	Climbing		5 days	Fibrinogen (g/L)	+23%	*p* < 0.025
Kicken et al. (20)	3883 (terrestrial)	11 M, 7 F (only 17 results) 21–50		Ascent by car and cable car	6 days	Hb (g/L)	+3%	*p* = 0.004
						Hct (L/L)	+9%	*p* = 0.0002
						F.VIII (%)	+8%	*p* = 0.0075
Kicken et al. (21)	3375 (terrestrial)	6 M 18–48	Vigorous exercise		6 days	Hb (g/L)	+24%	*p* < 0.0001
						vWF (%)	+53%	*p* = 0.0002
						vWF (%)	+59%	*p* = 0.0002
						F.VIII (%)	+43%	*p* = 0.0009
Lehmann et al. (22)	4559 (terrestrial)	10^b^	Climbing		18 h	PLT count (x 10^3^/μL)	−23%	*p* ≤ 0.001
Maher et al. (23)	4400 (simulated)	8 M 22 ± 1		Activities of daily living	48 h	Hct (L/L)^c^	+4%	*p* < 0.05
						PTT (sec)^c^	−370%^+^	*p* < 0.05
						Fibrinogen (g/L)^c^	+79%	*p* < 0.01
						F.VIII (%)^c^	−32%	*p* < 0.02
Martin et al. (24)	5300 (terrestial)	12 M, 5 F 34.0 ± 9.8	Climbing		17 days	Hb (g/L)	+20%	*p* < 0.01
Modesti et at. (25)	5400 (terrestrial)	25 M, 6 F 24–62	Climbing		19 days	Hct (L/L)	+19%	*p* < 0.001
Ninivaggi et al. (26)	3900 (terrestial)	14 M,16 F 28-32	7 M, 8 F Climbing	7 M, 8 F Ascent by cable car	8 days	Active: vWF (%)	+21%	*p* < 0.05
						Active: F.VIII (%)	+25%^a^	*p* < 0.05
						Passive: vWF (%)	+5%^+^	*p* < 0.05
						Passive: F.VIII (%)	+12%^+^	*p* < 0.05
Rocke et al. (28)	5200 (terrestrial)	37 M, 26 F 26.4 ± 3.9 G1: *n* = 41 G2: *n* = 22		Ascent by plane and car	7 days	G1: Hb (g/L)	+12%	*p* < 0.005
						G2: Hb (g/L)	+7%	*p* < 0.0001
						G1: Hct (L/L)	+14%	*p* < 0.005
						G2: Hct (L/L)	+17%	*p* < 0.0001
						G2: Fibrinogen (g/L)	+27%	*p* < 0.001
						G1: PLT count (x 10^3^/μL)	+19%	*p* < 0.005
						G1: PLT count (x 10^3^/μL)	+31%	*p* < 0.0001
Schaber et al. (29)	4500 (simulated)	21 M, 16 F 19–42		Free movement	12 h	PT (%)	+7%	*p* = 0.029
Singh et al. (33)	3505 (terrestial)	16 M 20–42		Free movement	3 days	Fibrinogen	−20%	*p* < 0.01
**Extreme altitude**								
Albrecht and Albrecht (14)	6200 (terrestrial)	G2 (placebo) 11^d^ (Age and sex NG)	Climbing		35 days	PT (%)	−46%	*p* < 0.001
Andrew et al. (15)	7625 (simulated)	8 M 21–31	Incremental cycling		40 days	F.VIII:Complex (%)	+40%	*p* < 0.05
Doughty et al. (19)	5700 (terrestrial)	6 M^e^	Climbing		48 days	Hb (g/L)	+44%	NG
						Bleeding time	+81%	NG
Pichler Hefti et al. (27)	6865 (terrestrial)	G1 (fast ascent): 15 M, 3 F G2 (slow ascent): 12 M, 4 F 46 (median)	Climbing		22 days	PT (%)	+16%^+^	*p* < 0.001
						aPTT (sec)	+38%^+^	*p* < 0.001
						APC-R	−16%	*p* < 0.001
						G1: vWF-RCo (%)	−24%^+^	*p* < 0.001
						G2: vWF-RCo (%)	−34%^+^	*p* < 0.001

*F, female; M, male; AG, antigen; APC-R, activated protein C resistance; aPTT, activated partial thromboplastin time; G1, group 1; G2, group 2; Hb, hemoglobin; Hct, hematocrit; μL, microliter; NG, not given; PLT, platelet; PT, prothrombin time; sec, second; vWF-Rco, von Willebrand factor-ristocetin cofactor activity. All p-values reflect intragroup comparisons unless otherwise indicated*.

a *At 2045 m*.

b *10 subjects out of 36 M and 4 F, aged 40 ± 9 yrs*.

c *Results after 1 h at altitude*.

d *Results after 1 h at altitude*.

e *Age and sex not reported*.

f *Age not reported*.

+ *Estimated from figures*.

† *Reported in the original study*.

Four studies were conducted in the Himalayan region (19, 25, 27, 34), three (14, 24, 28) in the Andean Mountains and two in the Italian Alps (16, 22). The other seven terrestrial trials did not specify their geographical setting (20, 21, 26, 30–33).

The remaining six studies used simulated altitude: two (15, 17) in a hypobaric setting, two (18, 29) in a normobaric setting, and one (23) in a normobaric and hypobaric hypoxia setting.

### Changes of Hemostasis at Moderate Altitude

A single study passively subjected 20 adults aged from 20 to 40 years to simulated altitude (17). After 8 h, increases of F.VIIa activity, F.VII antigen, prothrombin fragments 1 + 2 (PTF) and thrombin-antithrombin complex (TAT), as well as a small decrease of tissue factor pathway inhibitor (TFPI) activity and free antigen levels, have been shown.

### Changes of Hemostasis at High Altitude

In total, 16 studies including 422 subjects addressed this topic. Of these studies, 11 examined changes in association with physical activity, and another eight were concerned with passive hypoxic exposure (Tables 2–4). Only one study investigated the effects of passive and active hypoxia (26). Active exposures consisted primarily of trekking or mountaineering activities, with only occasional endurance exercise. The maximum duration of altitude (or simulated altitude) exposure varied from 12 h to 30 days. The age of the subjects ranged from 18 to 62 years, with 198 being male and 71 being female. In 52 persons, sex was not reported. The number of healthy subjects ranged from 6 to 76. Hemoglobin and hematocrit were shown to increase to differing extents; one study (28) observed an increased platelet count, while another (22) reported a decreased number. Regarding global coagulation, one study (23) demonstrated an increased PTT and another (29) an increased PT. F.VIII complex has been shown to increase with exercise and activities of daily living. One study (26) reported increased F.VIII activity, in contrast to the decrease observed by Maher (23). Three of fifteen studies demonstrated increased fibrinogen (16, 23, 28); only one reported a decrease (33). Two studies showed an increase in von Willebrand factor (vWF) and vWF antigen (21, 26). In regard to fibrinolysis, a decreased clot lysis time was seen in one study (33). Zafren and co-workers demonstrated negative (qualitative) D-dimer tests (34). Four studies applied viscoelastic tests. Two studies demonstrated increased clot strength (28, 29). Data on the speed of clot formation are inconsistent, with two studies (24, 25) reporting a prolonged initiation and another a short initiation (18).

One study showed more rapid increase in thrombin generation (TG) in whole blood after 8 days (26). Another study only observed an increase in TG after 6 days (20). Regarding platelet-rich plasma (PRP), one study showed an increased time to reach the peak (20) and decreased endogenous TG (ETP) after exercise (21). Two studies (22, 28) using the PFA-100 device (Siemens Healthineers, Erlangen, Germany) demonstrated decreased closure times, while another study (28) additionally using a platelet function analyzer (Multiplate; Roche Diagnostics, Mannheim, Germany) showed an increased area under the curve. Two studies (20, 21) reported decreased spontaneous and agonist-induced platelet activation as demonstrated by decreased levels of p-selectin and the fibrinogen αIIbβ3 receptor in 24 subjects. This is contrary to the increased p-selectin levels seen in another study (22). Sharma et al. observed increased platelet adhesiveness (30). Two further studies investigating platelet function showed no significant changes with altitude (31, 32).

### Changes of Hemostasis at Extreme Altitude

The four studies included on extreme altitude (*n* = 59) involved physical activity (mountaineering in three of them; Tables 2, 3). One study exposed the subjects to exhaustive cycling exercise at simulated altitude (15). The maximum duration of altitude/simulated altitude exposure varied from 22 to 48 days. Two of the studies reported participant age, which ranged from 21 to 31 years in the first of these studies (15) and had a median value of 46 years in the second (27). The studies included 41 males and 7 females. In 11 persons, sex was not reported. The number of subjects ranged from 6 to 34. The oldest study reported a nearly halved prothrombin time after 1 month (14). Operation Everest II demonstrated an increase in F.VIII complex after incremental bouts of cycling (15). In a later study, an increased hemoglobin and bleeding time after 48 days was seen (19). Lastly, after 3 weeks D-dimer concentrations, PT and aPTT all increased, while vWF-ristocetin cofactor activity (vWF-RCo) and activated protein C (APC) resistance decreased (27).

**Table 3:** Changes of markers for fibrinolysis during exposure to moderate, high and extreme altitudes (four studies, *n* = 146).

**References**	**Maximum altitude level [m]**	**Participants** **M/F, age [years]**	**Type of** **physical activity**		**Maximum duration**	**Parameter**	**Maximum change**	**Level of significance^†^**
			**Active**	**Passive**				
**Moderate altitude**								
Bendz et al. (17)	2,400 (simulated)	20 M 20–40		Daily live activity	8 h	TFPI activity (%) Free antigen (ng/mL)	−10% −17%	*p* = 0.001 *p* = 0.02
**High altitude**								
Singh et al. (33)	3,505 (terrestrial)	16 M 20–42		Free movement	3 days	Clot lysis time (h)	−29%	*p* < 0.01
Zafren et al. (34)	5,340 (terrestrial)	58 M, 18 F 20–62	Climbing		NG	D-dimer (qualitative)	negative	NA
**Extreme altitude**								
Pichler Hefti et al. (27)	6,865 (terrestrial)	G1 (fast ascent): 15 M, 3 F G2 (slow ascent): 12 M, 4 F 46 (median)	Climbing		22 days	G1: D-dimer (mg/L) G2: D-dimer (mg/L)	+93%^+^ +52%^+^	*p* < 0.001 *p* < 0.001

*F, female; M, male; h, hour; L, liter; μg, microgram; mL, milliliter; NA, not applicable; NG, not given; ng, nanogram; nM, nanomol; PTF, prothrombin fragments 1+2; TAT, thrombin-antithrombin complex; TFPI, tissue-factor-pathway inhibitor; U, units. All p-values reflect intragroup comparisons unless otherwise indicated*.

† *Reported in the original study*.

+ *Estimated from figures*.

**Table 4:** Changes of thromboelastometry, thrombin generation and platelet function during exposure to high altitude (ten studies, *n* = 210).

**References**	**Altitude level [m]**	**Participants** **M/F, age [years]**	**Type of** **physical activity**		**Maximum duration**	**Parameter**	**Maximum change**	**Level of significance^†^**
			**Active**	**Passive**				
**High altitude**								
Coppel et al. (18)	4,500 (simulated)	15 M, 13 F (only data from 25 subjects) 25 (median)		Activities of daily living	8 h	Thrombelastometry: Split time (sec) R time (sec)	–19% –19%	*p* = 0.022 *p* = 0.004
Kicken et al. (20)	3,883 (terrestrial)	11 M, 7 F (only data from 17 subjects) 21–50		Ascent by car and cable car	6 days	TG in WB: Peak height (nM) ETP (nM/min) TG in PRP: Peak height (nM) ETP (nM/min) Time to peak (min)	+28% +26% +23% +34% +10%	*p* = 0.0004 *p* = 0.0002 *p* = 0.0006 *p* < 0.0001 *p* = 0.03
						PLT activation (spontaneous): P-selectin (MFI) αIIbβ3 (MFI) PLT activation + ADP: P-selectin (MFI) αIIbβ3 (MFI) PLT activation + CRP: P-selectin (MFI) αIIbβ3 (MFI) PLT activation + TRAP: P-selectin (MFI) αIIbβ3 (MFI)	–23% –20% –30% –21% –14% –17% –14% –5%	*p* = 0.0031 *p* = 0.0032 *p* < 0.0001 *p* < 0.0001 *p* < 0.0001 *p* = 0.001 *p* < 0.0001 *p* < 0.0001
Kicken et al. (21)	3,375 (terrestrial)	6 M 18–48	Vigorous exercise		6 days	TG in PRP ETP (nM/min) TG in PPP after addition of high dose of TF: ETP (nM/min)	–58% +11%	*p* = 0.003 *p* = 0.03
						PLT activation + CRP: P-selectin (MFI)^b^ αIIbβ3 (MFI)^a^ P-selectin (MFI)^b^ αIIbβ3 (MFI)^b^ PLT activation + TRAP-6: P-selectin (MFI)^a^ αIIbβ3 (MFI)^a^ P-selectin (MFI)^b^ αIIbβ3 (MFI)^b^	–615^#^ –1.305^#^ –1.436^#^ –1.610^#^ –665^#^ –423^#^ –1.351^#^ –498^#^	NS *p* < 0.05 *p* < 0.05 *p* < 0.05 NS *p* < 0.05 *p* < 0.05 *p* < 0.05
Lehmann et al. (22)	4,559 (terrestrial)	36 M, 4 F (only data from 10 controls) 40 ± 9	Climbing		18 h	PLT function (PFA-100): Closure time + ADP (sec) Closure time + EPI (sec) PLT activation (spontaneous): P-selectin (ng/mL)	–29% –31% +277%	*p* ≤ 0.001 *p* ≤ 0.001 *p* < 0.001
Martin et al. (24)	5,300 (terrestrial)	12 M, 5 F 34.0 ± 9.8	Climbing		17 days	Thrombelastometry: R-time (sec) K-time (sec) α-angle (degree)	+30% +207% –30%	*p* = 0.016 *p* < 0.001 *p* < 0.001
Modesti et at. (25)	5,400 (terrestrial)	25 M, 6 F 24–62	Climbing		19 days	InTEM CT (12d)	+10	*p* = 0.003
						InTEM CFT (12d)	+20%	*p* = 0.014
						InTEM alpha (12d)	–5%	*p* = 0.012
						InTEM MAXV (12 +20d)	–18%	*p* < 0.001
Ninivaggi et al. (26)	3,900 (terrestial)	16 M, 14 F 28–32	G1: 7 M, 8 F Climbing	G2: 7 M, 8 F Ascent by cable car	8 days	TG in WB: G 1 (active): Peak height (nM) ETP (nM/min) Lag time (sec) Time to peak (sec) Velocity index	+23% +18% –30% –45% +50%	*p* < 0.05 *p* < 0.05 *p* < 0.05 *p* < 0.05 *p* < 0.05
						G2 (passive): Peak height (nM) ETP (nM/min) Lag time (sec) Time to peak (sec) Velocity index	+50% +38% –33% –29% +50%	*p* < 0.05 *p* < 0.05 *p* < 0.05 *p* < 0.05 *p* < 0.05
Rocke et al. (28)	5,200 (terrestrial)	12 M, 10 F (only data from Apex 4 available) 26.4 ± 3.9		Ascent by plane and car	7 days	Thrombelastometry: FibTEM A20 (mm) ExTEM A20 (mm)	+22% +6%	*p* < 0.01 *p* < 0.01
						PLT function (Multiplate): ADP AUC (AU)	+22%	*p* < 0.01
						PLT function (PFA-100): Closure time + EPI	–50%	*p* < 0.00001
Schaber et al. (29)	4,500 (simulated)	21 M, 16 F (only data from 14 subjects) 19–42		Free movement	12 h	Thrombelastometry InTEM CT (sec) InTEM MCF (mm) FibTEM MCF (mm)	–4% +4% +12%	*p* = 0.012 *p* = 0.039 *p* = 0.035
Sharma (30)	3,000 (terrestrial)	15 M 21–26		Activities of daily living	21–30 days	Platelet adhesiveness (%)	+14%	*p* < 0.001

*F, female; M, male; ADP, adenosine diphosphate; AU, aggregation units; AUC, area under the curve; αIIbβ3, fibrinogen αIIbβ3 receptor; Cmax, maximum concentration of thrombin; CRP, collagen-related peptide; CFT, clot formation time; CT, clotting time; EPI, epinephrine; ETP, endogenous thrombin potential; G group; GXT, graded exercise test; HAPE, high altitude pulmonary edema; MAXV, maximum velocity; MFI median fluorescence intensity expressed in arbitrary units; min, minute; mm, millimeter; nM, nanomol; PFA, platelet function analysis; PLT, platelet; PPP, platelet-poor-plasma; PRP, platelet-rich plasma; R-time, reaction time; ROTEM, rotational thromboelastometry; sec, second; SP, split point; TF, tissue factor; TG, dynamic thrombin generation; tPA, tissue plasminogen activator; TRAP-6, thrombin receptor-activating peptide; WB, whole blood. All p-values reflect intragroup comparisons unless otherwise indicated. NS, not significant*.

a *Results on 6th day 2 h before exercise*.

b *Results on 6th day 2 h after exercise*.

# *Changes expressed as absolute values*.

† *Reported in the original study*.

### Risk of Bias Within Studies

The multitude of protocols applied by the included studies limits comparability; for example, normobaric hypoxia and weather in a terrestrial setting represent potential confounders. The absence of blinding of participants and personnel in most of the studies may represent another source of bias. As the data of a few studies had to be estimated by manual measurements, the accuracy of the data may have been compromised. Lastly, the studied subjects were fairly young adults and predominantly male (only 102 females out of 501 subjects). These limitations have to be kept in mind when interpreting the results.

## Discussion

A multitude of data on changes of hemostasis induced by hypoxic exposure, with a specific focus on prothrombotic effects, exist. However, whether there is scientific evidence of a causal relationship between hypoxic exposure, either at real or simulated altitudes, and thrombosis in healthy subjects is still a matter of debate.

### Moderate Altitude and Hemostasis

At moderate altitude a single study demonstrated activation of coagulation after short-term hypobaric hypoxia, as shown by an increase in PTF and TAT (17). Both of these compounds are produced directly by thrombin activation and have been shown to indicate a risk of thrombosis (37). In addition, markers of fibrinolysis did not change during hypoxic chamber exposure. Thus, at least for moderate hypoxic conditions, there is a lack of evidence that exposure to moderate altitudes is associated with thrombogenic conditions.

### High Altitude and Hemostasis

Altitude-related hemoconcentration is a phenomenon during high-altitude exposure due to a plasma volume reduction in the early phase of adaptation, along with increased erythropoiesis after several weeks and the development of polycythemia. Hemoconcentration is suggested to affect hemostasis (38). Moreover, with increasing hematocrit levels, platelets are increasingly concentrated near walls *in vitro* (39, 40). Our literature search revealed five trials (20, 21, 23, 25, 28) reporting slightly increased hemoglobin and hematocrit levels, while one study (16) showed no change in either parameter. Regarding global coagulation tests, the aPTT and PT results were inconsistent, with a pronounced shortening of aPTT reported after short-term hypoxia that then returned to baseline (23), along with no change of aPTT (16) or a small increase after several hours of hypoxia (29). As neither test truly reflects *in vivo* coagulation activity, these findings are of limited value (41). Fibrinogen plays a pivotal role in the formation of a stable clot. In contrast to a single study reporting a small drop of fibrinogen after 3 days at high altitude (33), other studies observed either slightly elevated fibrinogen levels during high-altitude exposure (16, 28) or an early increase with normalization thereafter (23). Fibrinogen remained unchanged in another study involving passive exposure (20). VWF acts as a procoagulant, mediating the adhesion of platelets to damaged vessel walls via binding both to the collagen and platelet receptor. Moreover, it mediates the protection conferred by procoagulant factor VIII. An increased plasma level is associated with a higher risk of venous thrombosis (42). In our review, vWF antigen and F.VIII showed similar reactions; VWF was increased with active exposure and exercise (21, 26), while in subjects ascending passively, hypoxia *per se* led to either unchanged or only minor increased levels (20, 21, 26). F.VIII activity increased to a differing extent (20, 21, 26). Greater increases were observed after strenuous exercise performed at high altitude (21) and during active ascent compared to passive ascent (26, 28). Passive ascent is associated with only minor increases in F.VIII activity (20, 21), or even with unchanged levels (16). In summary, hypoxia *per se* seems to increase vWF and F.VIII only slightly; physical exercise may promote a greater increase.

Two markers of thrombin activation, PFT and TAT, remained unchanged in one study (22). Newer data showed elevated TG in whole blood, but not in plasma (26). The same group reported a slight increase of F.VIII-mediated TG in whole blood in healthy inactive volunteers (20), which was balanced by decreased platelet activation. With vigorous exercise, TG in whole blood increased slightly upon arrival at a high-altitude area (and after 2 days) in platelet-poor-plasma (PPP), with a concomitant decrease of platelet activation and platelet-dependent TG (21).

Data regarding fibrinolysis largely showed a lack of changes in related parameters, such as the clot lysis time (21, 26), D-dimer level according to qualitative (34) or quantitative assay (20), and antithrombin (15, 20), alpha-2-macroglobulin, plasminogen and PC levels (15). In summary, these data support that fibrinolysis is only moderately affected by high-altitude hypoxia.

In the past few decades, platelets and platelet functions have been recognized to play a crucial role in hemostasis. Results for platelet count range from a short-term reduction after 1 day (22) to small increases after 1 week (28). All changes were within the normal laboratory ranges. With respect to platelet activity, increased platelet adhesiveness (22, 30), and platelet activation and sequestration, were reported (22). In contrast, Kicken et al. observed lower spontaneous platelet activation at high altitude compared to sea level (20). The authors stated that hypercoagulability, as reflected in the increased factor VIII-mediated TG, was balanced by decreased platelet activation. Data from the same group demonstrated a slight decrease in platelet granule release potential, and a decrease in platelet aggregation potential and platelet-dependent TG, at high altitude (21). Rocke at al. observed increased platelet reactivity, concluding that this indicates a hypoxia-induced increased in platelet reactivity, and thus a prothrombotic phenotype at altitude (28). Recently, a change in the platelet proteome in extreme hypoxia conditions (>7,000 m) was demonstrated by increased platelet reactivity in association with enhanced calpain activity in a murine model (43).

The results of viscoelastic methods are contradictory. Passively exposing 25 participants to normobaric hypoxia shortened clot initiation after 8 h (18). However, the clot formation time was still within the normal range. On the contrary, Martin et al. reported dysfunctional clot formation despite increased hemoglobin after 2 weeks (24). Modesti and co-workers reported a prolonged clot formation of the intrinsic pathway ranging within normal values (25). Schaber and co-workers demonstrated a shortening of the coagulation time and increased clot strength after 12 h, again within normal ranges (29). Lastly, Rocke showed increased clot strength after 1 week (28). However, the relationship between increased clot strength and thrombosis risk was assumed only in a high-risk surgical setting (44), in the context of recurrent ischemic events after percutaneous coronary intervention (45) and after ischemic stroke (46). Therefore, results obtained by viscoelastic methods do not support increased thrombogenicity at high altitudes within 1 week in healthy individuals traveling from low altitudes.

### Extreme Altitude and Hemostasis

Studies on extreme altitudes in the literature were limited, so the influence of these altitudes on the hemostatic system is not clear. PT was demonstrated to either decrease or mildly increase after 5 weeks (14). A prolonged bleeding time, as measured by the Ivy technique after 3 weeks, is of limited value as this method is poorly reproducible (19). Recently, a prolonged aPTT in combination with PC inactivation and decreased vWF-RCo with increasing altitudes have been shown (27). The authors explained these results in terms of increased consumption of vWF due to the activated fibrinolysis. Simulated conditions of cycling exercise to exhaustion were associated with an increased F.VIII complex (15). In summary, the available data demonstrate that activation of coagulation is associated with fibrinolysis stimulation at extreme altitude. However, whether this is attributable to exercise or hypoxia *per se* is still unclear.

### Underlying (Pathophysiological) Responses to Environmental Hypoxia

Essential for the cellular response to hypoxia is an increased stimulation of hypoxia-inducible factor (HIF) and a consequent expression of a great variety of genes (1, 47). Acute adaption to hypoxia is regulated mainly via HIF-1 which stimulates erythropoiesis and angioneogenesis. In high-altitude dwellers a less pronounced erythrocytosis, a lower pulmonary arterial blood pressure and a higher SaO2 compared to lowlanders are suggestive of this adaptive process (48). One of the pivotal changes includes a shift toward a greater HIF-2 and HIF-3 expression. Geographical differences of the same populations exist. In Tibetans a variation of EPAS1 (which encodes HIF) has been shown to be associated with lower hemoglobin levels and a decreased response to pulmonary vasoconstriction. Tibetans living at sea level have adapted by a variation of EPAS1 and a consequent hyporesponsive HIF transcriptional system (47, 49). Chronic exposure to high altitude can further result in different changes in the hematological system. Data from acquired diseases affecting hemostasis such as polycythemia vera point toward a chronic augmentation of HIF during chronic hypoxic exposure (13). Polycythemia vera in patients residing at high altitude has been shown to be associated with an increased the risk of thrombotic complications (13).

With regard to coagulation, human platelets from healthy probands have been shown to upregulate expression of HIF-2a and the antifibrinolytic PAI-1 after hypoxic exposure *in vitro* (50).

Despite these informations on the involvement of HIF in hypoxic adaptation, the significance of such a response on the hemostatic system for acute adaptation and short term exposure at high altitude remains to be investigated.

### Strength and Limitations

One strength of our systematic review was that we only included studies providing data on healthy persons. Thus, confounding effects of pre-existing diseases can be ruled out. However, hereditary disorders of coagulation (e.g., APC resistance, PC or PS deficiency, prothrombin-complex polymorphism, etc.) were not analyzed in the selected studies at baseline, such that subjects with these conditions may have been included.

Another strength, and a novelty, of this study was our analysis of the degree of hypoxia according to altitude (moderate, high or extreme), and the sub-categorization of active and passive ascent and sojourns. Sojourns at altitude are mostly associated with physical exercise, differing in intensity and duration. However, since none of the selected studies described the exercise habits of their participants in detail, a definitive conclusion on the additive effects of hypoxia and exercise on coagulation cannot be drawn. At sea level, moderate-intensity sport does not cause hypercoagulability in healthy persons or those with lifestyle diseases. However, very intensive exertion significantly activates coagulation and platelets, and concomitantly enhances fibrinolysis, with rebalanced hemostasis seen during exercise. In contrast to hypercoagulability, which can last for several hours after exercise, fibrinolytic parameters return to baseline much earlier (51). This loss of balance immediately after strenuous exercise is suggested to be critical for persons with cardiovascular diseases and could trigger cardiovascular events (52, 53). This knowledge may be important for mountaineers and trekkers with known pre-existing cardiovascular diseases (e.g., hypertension). Since no data on the incidence of cardiovascular diseases and adverse cardiovascular events in mountaineers/trekkers at high altitudes are available, the relation between strenuous exercise under hypobaric hypoxic conditions and venous thromboembolic events (e.g., VTE, CVST, etc.) and arterial thrombosis (e.g., myocardial infarction, stroke, etc.,) needs to be investigated.

Reductions of plasma volume and hemoconcentration is a phenomenon seen early during high-altitude adaptation. Hypoxia increases red cell mass after a few weeks, leading to high-altitude polycythemia (54). Coldness, gusty winds and a high exercise load during mountaineering could further promote loss of plasma volume, leading to dehydration. However, data on the *in-vivo* effect of polycythemia/dehydration upon hemostasis remain equivocal. Viscoelastic tests revealed decreased clot strength with increasing amounts of red blood cells (RBCs), but this was accompanied by enhanced platelet aggregation and an increased platelet count (55). Moreover, an increased RBC count increases the endogenous thrombin potential (56). On the other hand, *in-vitro* hypercoagulability has been shown to be associated with low hematocrit values (57). Moreover, *in-vitro* studies showed that platelets were increasingly concentrated near walls with increases in hematocrit (39, 40). This may be counteracted by a decreased platelet aggregation potential at high altitude (21). To further complicate the issue, only viscoelastic and platelet function tests measure increases in hematocrit.

Sympathoadrenergic activation plays a central role in high-altitude adaptation (58). In contrast to moderate altitudes, at high and extreme altitudes the adrenergic drive persists during the entre sojourn, which is reflected in high catecholamine concentrations. Physical and psychological stress (e.g., bad weather/storms, snowfall, cold conditions, sitting in a tent with limited space, etc.,) can exacerbate adrenergic activation. Stress responses could induce a prothrombotic state characterized by autonomic and neuroendocrine dysfunction, platelet activation, dysregulation of coagulation, fibrinolysis and endothelial dysfunction (59). Since none of the studies included in this review reported such extreme conditions, it cannot be excluded that thrombogenicity is more likely under such conditions.

To rule out sex-related differences, many studies included only male volunteers. As women represented only around one quarter of all subjects, and are known to show a different hemostatic response to exercise (60, 61), the influence of gender on outcomes needs to be addressed in further studies.

One limitation of the studies included in this review is the fact that, in the older studies, the parameters measured for coagulation, fibrinolysis and platelet function are of limited methodological value for the interpretation of our findings. In addition, most studies only analyzed parts of the hemostatic system, making interpretation of the influence of hypoxia on hemostasis difficult.

### Future Directions

Future trials should differentiate between the hemostatic responses to hypoxia *per se* and to repetitive exercise in hypoxia. These studies should include evenly distributed male and female subjects and correct for plasma volume changes. As all measurements are *in vitro* assays, newer technologies for coagulation monitoring could provide a deeper inside into the complex interplay of the hemostatic response to profound hypoxia *in vivo*.

Since the high number of trekkers and trekking tourists at high altitude remote areas we need more information on their health status before arrival and more data on adverse health outcomes during and after the tours. Thus, in order to elucidate the role of hypoxia on hemostasis, a multicenter, multinational registry is needed including data on pre-existing cardiovascular diseases and acute thrombotic and thromboembolic events.

## Conclusion

At all levels of hypobaric hypoxia, i.e., from moderate to extreme altitudes, changes in the various parameters of coagulation, platelet function and fibrinolysis have been reported in the literature. However, due to the multiplicity of confounding factors at altitude (e.g., training status, duration and intensity of exercise, fluid status, mental stress, etc.,), there is no consensus on the role of hypoxia alone in these changes. Referring to a hypothesis suggested in the literature, hypoxia itself could be the trigger for a thrombogenic state. Therefore, we cannot support the assumption that hypoxia occurring in mountainous regions is the primary trigger for hypercoagulability and an increased risk of thromboembolic disorders, at least in healthy persons.

## Data Availability Statement

The original contributions presented in the study are included in the article/Supplementary Material, further inquiries can be directed to the corresponding author.

## Author Contributions

WS and DF: conceptualization of the study. WS and CB: methodology. BT, BW, WS, and CB: data analysis. WS, CB, and DF: supervision. BT, BW, and WS: writing—original draft. BT, BW, WS, CB, and DF: writing—review and editing. All authors contributed to the article and approved the submitted version.

## Conflict of Interest

CB and WS were employed by Private University for Health Sciences, Medical Informatics and Technology UMIT, Hall i.T. and Tirol Kliniken GmbH. The remaining authors declare that the research was conducted in the absence of any commercial or financial relationships that could be construed as a potential conflict of interest.

## Publisher's Note

All claims expressed in this article are solely those of the authors and do not necessarily represent those of their affiliated organizations, or those of the publisher, the editors and the reviewers. Any product that may be evaluated in this article, or claim that may be made by its manufacturer, is not guaranteed or endorsed by the publisher.

## Supplementary Material

Supplementary Table S1 Oxford Level of Evidence scale and modified Downs and Black Quality Assessment results.

## Abbreviations

APC: activated protein C
CVST: cerebral venous sinus thrombosis
HACE: high-altitude cerebral edema
HAPE: high-altitude pulmonary edema
HIF: hypoxia inducible factors
PAE: pulmonary arterial embolism
PTF: prothrombin fragments 1 + 2
PC: protein C
PS: protein S
RBC: red blood cell
ROTEM: rotational thrombroelastometry
TAT: thrombin-antithrombin complex
TEG: thrombelastography
TFPI: tissue factor pathway inhibitor
TG: thrombin generation
VWF-RCo: von Willebrand Factor-ristocetin cofactor activity
VTE: venous thromboembolism.
