# Supplementary material for: The Influence of Environmental Hypoxia on Hemostasis—A Systematic Review
Source: Front Cardiovasc Med. 2022 Feb 18;9:813550. doi: 10.3389/fcvm.2022.813550 (PMC8894865; doi:10.3389/fcvm.2022.813550)
Supplement: Supplementary Table S1 — Oxford Level of Evidence scale and modified Downs and Black Quality Assessment results. [file Table_1.DOCX]

Supplementary Material

Table S1 | Oxford Level of Evidence scale and modified Downs and Black Quality Assessment results.

|  |  | |  | |  | | | |  |
| --- | --- | --- | --- | --- | --- | --- | --- | --- | --- |
| **Authors** | **Level of Evidence** | | **Quality assessment score** | | | **Quality rating** | |  |  |
|  |  |  | |  | | |  | | |
|  |  | |  | | |  | |  |  |
| Albrecht E and Albrecht H. (1969) | 2b | | 7 | | | Fair | |  |  |
|  |  | |  | | |  | |  |  |
| Andrew M et al. (1987) | 4 | | 5 | | | Poor | |  |  |
|  |  | |  | | |  | |  |  |
| Bartsch P et al. (1989) | 4 | | 7 | | | Fair | |  |  |
|  |  | |  | | |  | |  |  |
| Bendz B et al. (2000) | 2b | | 6 | | | Poor | |  |  |
|  |  | |  | | |  | |  |  |
| Coppel J et al. (2019) | 2b | | 3 | | | Poor | |  |  |
|  |  | |  | | |  | |  |  |
| Doughty H and Beardmore C. (1994) | 4 | | 2 | | | Poor | |  |  |
|  |  | |  | | |  | |  |  |
| Kicken C et al. (2018) | 2b | | 8 | | | Fair | |  |  |
|  |  | |  | | |  | |  |  |
| Kicken C et al. (2019) | 2b | | 8 | | | Fair | |  |  |
|  |  | |  | | |  | |  |  |
| Lehmann T et al. (2006) | 2b | | 4 | | | Poor | |  |  |
|  |  | |  | | |  | |  |  |
| Maher J et al. (1976) | 4 | | 4 | | | Poor | |  |  |
|  |  | |  | | |  | |  |  |
| Martin D et al. (2012) | 2b | | 7 | | | Fair | |  |  |
|  |  | |  | | |  | |  |  |
| Ninivaggi M et al. (2015) | 1b | | 9 | | | Good | |  |  |
|  |  | |  | | |  | |  |  |
| Pichler Hefti J et al. (2010) | 1b | | 9 | | | Good | |  |  |
|  |  | |  | | |  | |  |  |
| Rocke A et al. (2018) | 2b | | 8 | | | Fair | |  |  |
|  |  | |  | | |  | |  |  |
| Schaber M et al. (2015) | 2b | | 10 | | | Good | |  |  |
|  |  | |  | | |  | |  |  |
| Sharma S and Hoon R. (1978) | 4 | | 4 | | | Poor | |  |  |
|  |  | |  | | |  | |  |  |
| Sharma S. (1981) | 4 | | 4 | | | Poor | |  |  |
|  |  | |  | | |  | |  |  |
| Sharma S. (1982) | 4 | | 5 | | | Poor | |  |  |
|  |  | |  | | |  | |  |  |
| Singh I et al. (1969) | 4 | | 4 | | | Poor | |  |  |
|  |  | |  | | |  | |  |  |
| Zafren F et al. (2011) | 2b | | 8 | | | Fair | |  |  |
|  |  | |  | | |  | |  |  |

Level of evidence: 1b, individual RCT with narrow confidence limits; 2b, exploratory cohort study; 4, poor quality cohort or case-control study. Scoring criteria: 11-13: Excellent; 9-10: Good; 7-8: Fair; 0-6: Poor.
